# Micro Spectroscopic Photoacoustic (μsPA) imaging of advanced carotid atherosclerosis

**DOI:** 10.1016/j.pacs.2021.100261

**Published:** 2021-03-18

**Authors:** Sophinese Iskander-Rizk, Mirjam Visscher, Astrid M. Moerman, Suze-Anne Korteland, Kim Van der Heiden, Antonius F.W. Van der Steen, Gijs Van Soest

**Affiliations:** Department of Cardiology, Erasmus MC University Medical Center Rotterdam, Rotterdam, the Netherlands

**Keywords:** μsPA, Micro Spectroscopic Photoacoustic, CE, cholesteryl ester, CEA, carotid endarterectomy, DHB, 2,5-dihydroxybenzoic acid, DG, diacylglycerol, ESI, electrospray ionization, HPLC, high-performance liquid chromatography, FTICR, fourier-transform ion cyclotron resonance, MALDI-MSI, matrix-assisted laser desorption ionization mass spectrometry imaging, *m/z*, mass to charge ratio, NIRS, near-infrared spectroscopy, PC, phosphatidylcholine, PCA, principal component analysis, PFA, paraformaldehyde, SM, sphingomyelin, TG, triacylglycerol, WREnS, Waters Research Enabled Software suite, Microscopy, Spectroscopy, Mass spectrometry imaging, Lipids, Atherosclerosis, PCA

## Abstract

Atherosclerosis is a lipid-driven and an inflammatory disease of the artery walls. The composition of atherosclerotic plaque stratifies the risk of a specific plaque to cause a cardiovascular event. In an optical resolution photoacoustic microscopy setup, of 45 μm resolution, we extracted plaque lipid photoacoustic (PA) spectral signatures of human endarterectomy samples in the range of 1150–1240 nm, using matrix assisted laser desorption ionization mass spectrometry imaging as a reference. We found plaque PA signals to correlate best with sphingomyelins and cholesteryl esters. PA signal spectral variations within the plaque area were compared to reference molecular patterns and absorption spectra of lipid laboratory standards. Variability in the lipid spectroscopic features extracted by principal component analysis of all samples revealed three distinct components with peaks at: 1164, 1188, 1196 and 1210 nm. This result will guide the development of PA-based atherosclerosis disease staging capitalizing on lipidomics of atherosclerotic tissue.

## Highlights

• We built a micro-spectroscopic photoacoustic imaging setup, allowing spectral imaging throughout the section.
• Spectral differences were primarily due to differences in hydrocarbon chain length and number of unsaturated bonds.
• PA signal intensity was most strongly correlated with sphingomyelin and cholesteryl ester area in MALDI-MSI.
• The spectral feature of lipids abundant in necrotic core can be used for detection of atherosclerosis with features of instability.

## 1 Introduction

Cardiovascular diseases, predominantly associated with advanced atherosclerosis, account for about 50 % of deaths worldwide and are a major cause of population morbidity [1,2]. Atherosclerosis gradually progresses over time; substances in the blood, such as lipids and inflammatory cells, are deposited at sites of dysfunctional endothelium, accumulating into the arterial wall and forming so-called plaques. These plaques may narrow the vessel, hindering blood flow. Unstable plaques can rupture; contact between plaque contents and blood can cause a thrombus to form, interrupting the blood supply to organs causing lethal events such as a Stroke or Myocardial Infarction.

The development of methods for monitoring the progress of atherosclerosis to detect and predict unstable plaque formation is an active research area which aims to prevent acute ischemic events, thereby reducing the disease burden of atherosclerosis [[3], [4], [5], [6]]. Hypotheses regarding the development of the disease to unstable lesion types and triggers for rupture have been formed and reformed over time, describing atherosclerosis as a multi-factorial inflammatory disease [[7], [8], [9], [10], [11], [12], [13]]. Atherosclerotic plaques are classified into different stages based on plaque composition and size with histology as a golden standard for assessment [14]. In autopsy studies of sudden cardiac death cases [14], the plaque phenotype which caused most thrombotic events has been described as a large lipid-rich necrotic core contained by a thin fibrous cap, named a thin-cap fibroatheroma.

Lipids are involved in all stages of the disease. Lesions first appear as a fatty streak phenotype, which consists of accumulated smooth muscle cells and macrophages having internalized oxidized lipoproteins. The advanced stage includes a lipid-rich necrotic core, with a very heterogeneous lipid composition [[15], [16], [17]]. Previous research has studied the relation between plaque phenotype and evolution on one hand, and lipid composition on the other [16,18,19]. This opens the perspective of lipid typing of plaques in-vivo, which could potentially be applied for diagnosis or prognosis, and used to guide treatment decisions in patients with coronary and carotid atherosclerosis. Current imaging modalities enabling atherosclerosis-related lipid identification in-vivo are low resolution MRI [20,21], and catheter-based near-infrared spectroscopy (NIRS) [22], which is clinically available, and catheter-based photoacoustic (PA) imaging [23]. NIRS, in its commercial realization, derives the probability of the presence of lipid-core plaque [[24], [25], [26]] based on detection of scattered light, and does not provide depth information. PA imaging, on the other hand, probes tissue molecular composition in depth and thus can create images of tissue type. In addition, it can be straightforwardly integrated with ultrasound imaging to deliver morphological information and plaque sizing. Spectroscopic photoacoustic imaging was previously shown to distinguish between peri-adventitial and plaque lipids, not only owing to its capability of depth resolution, but also through sensitivity to differences in absorption spectra of lipid types [27]. Spectral differentiation of different cholesteryl compounds, namely cholesterol, cholesterol linoleate and cholesterol oleate could be achieved with PA imaging [28,29], showing the potential of the modality to identify lipid profiles classifying plaques as stable and unstable.

In this study we extract the lipid signature from human carotid endarterectomy samples, in the range of 1150–1240 nm using an in-house developed Optical Resolution Photoacoustic Microscopy (OR-PAM) system capable of spectral imaging, that we refer to as micro-spectroscopic PA (μsPA), with images of lipid composition acquired by matrix-assisted laser desorption ionization mass spectrometry imaging (MALDI-MSI) as a reference. This is the first work, to our knowledge, which explores lipid PA spectral signatures and their distribution within advanced plaques at a microscopic level with corresponding molecular validation. Findings from this study can possibly be extrapolated for generation of optimal contrast in non-invasive carotid PA imaging or catheter-based coronary intravascular PA (IVPA) imaging.

## 2 Material & methods

### 2.1 Tissue collection and processing

Six human carotid endarterectomy (CEA) samples were surgically removed and snap frozen and stored at ↙80 °C until further processing. Tissue collection was performed according to Erasmus MC Ethics Board protocols (MEC 2008-147). The surgical protocol used preserves plaque morphology and intact lumen [30]. CEAs were transversally divided into 2 mm thick cross-sections, which were subsequently embedded into 10 % porcine type A gelatin (Sigma-Aldrich, The Netherlands). CEA cross-sections were cryo-sectioned into 10 μm thick sections and thaw-mounted onto glass slides, and stored at ↙80 °C. One cryosection was used for MALDI-MSI and an adjacent cryosection was used for μsPA, after the measurement the sections were histochemically stained for co-registration purposes.

### 2.2 Micro-spectroscopic photoacoustic imaging

#### 2.2.1 Experimental setup

**Fig. 1:**
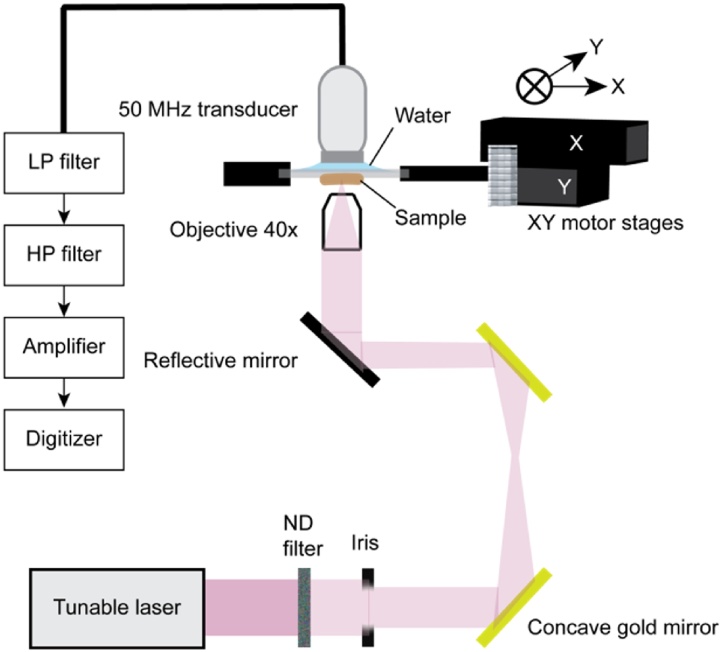
Experimental setup of micro spectroscopic photoacoustic imaging.

A micro-spectroscopic photoacoustic imaging (μsPA) setup (Fig. 1) was built by focusing the beam from a tunable diode pumped laser OPO (Spitlight EVO-OPO, 200 Hz PRF, 5 ns pulse width, Innolas GmbH, Germany) achromatically to a 45 μm spot, which we chose to match the resolution of the MALDI images. Pulse-to-pulse laser intensity variations were monitored using a photodiode. On average, the fluence varied between 300μJ/cm^2^ to 500 μJ/cm^2^ depending on the wavelength. An unfocussed 50 MHz transducer (V358-SU, Panametrics-NDT, USA) was spatially aligned with the optical beam to acquire transmission mode photoacoustic signals. A standard microscope slide (thickness 1 mm) with the tissue section was spatially raster scanned by two motor stages (Newport Corporation, USA). One motor was scanned, meanwhile allowing for a 4-fold averaging within the 45 μm step size, while the other motor was used to step 45 μm between scanlines. Photoacoustic signals were filtered by a low pass filter with cutoff 70 MHz (BLP-70, Mini-Circuits,) and high pass filtered with cutoff 25 MHz (BHP + 25, Mini-Circuits) and amplified 55 dB (AU-3A-0110, Miteq, Long Island, NY, USA) then digitized at 400 MHz (DP310, Aquiris).

#### 2.2.2 Tissue measurements

Prior to imaging, tissue cryosections were fixated by submersion into 4% paraformaldehyde (PFA) for 10 min [31,32]. A photoacoustic image of the complete tissue section was acquired at 1210 nm after which a region of interest (ROI) was chosen for spectroscopic imaging. During spectroscopic imaging the wavelength was swept from 1150–1240 nm in steps of 2 nm. Depending on the size of the endarterectomy sample cross-section the 1210 nm scan could take up to 25 min, while the spectroscopic scan of the sub-region could take up to 6 h.

#### 2.2.3 Lipid extract measurements

Six lipid extracts: a phosphatidylcholine PC(16:0/18:1) (850457C-25 mg, Avanti Polar Lipids, USA), two sphingomyelin species SM(d18:1/16:0) (1 mg, 6254-89-3, Cayman Chemical, USA), SM(d18:1/24:1) (860593P-5 mg, Avanti Polar Lipids, USA), a triacylglycerol TG(18:1/18:1/16:0) (2190-30-9, 10 mg, Sigma-Aldrich, The Netherlands), cholesteryl ester (CE) linoleate, CE(18:2) (700269P-100 mg, Avanti Polar Lipids, USA), and its oxidized form, CE ox-18:2 (700192P-500 μg, Avanti Polar Lipids, USA) were purchased. Lipids purchased in powder form were dissolved in 1 mL HPLC-grade chloroform. Lipids were deposited onto a glass slide and the chloroform was left to evaporate, leaving a lipid deposit on the slide. Spectroscopic imaging was performed using the same protocol as used for the tissue measurements. In order to assess the effect of fixation and gelatin embedding on the results, we additionally measured multiple CE18:2 prepared slides under all possible combinations of the following two conditions: (1) fix the slide in 4% paraformaldehyde (PFA) for 10 min, (2) deposit lipid on gelatin cryo-sections.

#### 2.2.4 Data processing

μsPA data was processed by taking the maximum intensity projection in two time windows of the analytical signal per pixel (absolute Hilbert) to generate two photoacoustic images. The tissue optical absorption image was generated using a square window corresponding to the time of flight of the acoustic signal through the glass slide. A tissue optical transmission image was generated using a square window in the PA signal trace, with a timing corresponding to the transducer response to light absorption in its surface [33]. Maximum intensity projection images were median filtered and convolved with a circular filter to account for the beam circular shape remapped to square pixels spatially. Signal intensity was corrected for laser pulse-to-pulse variations and filtered by a Savitsky-Golay filter of order 1 and frame length 5 all using MATLAB 2019b (Natick, MA, USA). Photoacoustic spectra of lipid extracts were processed similarly, averaging all spectra over the drop area to yield a standard spectrum per lipid standard. Selected images and spectra displayed in figures are normalized to the 95th percentile of the selected set.

Spectral variation in the data was extracted using principal component analysis (PCA). To ensure high quality spectral information, data was normalized to the noise level of every individual tissue section and masked using a threshold at 15 % above the mean noise level. The number of components shown was determined using the elbow-method, retaining only the statistically significant components. Spatial distribution of the PCA components is depicted using diverging color scales, limits of the color scale are set to the smallest absolute median value of a certain PCA component, and the scaling is symmetrical. The overlay image is a sum of all three components scaled from -0.5 to 0.5 combined.

### 2.3 MALDI-MSI

#### 2.3.1 Measurements

Samples were prepared for MALDI-MSI by sublimation of 50 mg 2,5-dihydroxybenzoic acid (DHB) onto the tissue using a home-built sublimation system [34]. The protocol uses 50 mg of DHB dissolved in 5 mL of aceton, sublimated at 125 °C for 10 min. MALDI-MSI was performed using a Synapt G2Si-TOF system (Waters, Manchester, UK) operated in the systems resolution mode. The spatial resolution of the experiment was 45 Ô 45 μm^2^, using a 2000 Hz Nd:YAG (355 nm) laser, 100 laser shots per pixels and a mass range of 300-1200 *m/z*. Data was acquired using Waters Research Enabled Software suite (WREnS) and MassLynx v4.2 and exported to imzML format using HDI v1.4.

*M/z* values were identified as lipid species by means of FTICR MALDI-MSI (Bruker Daltonics, Bremen, Germany) uploading the data to METASPACE annotation platform [35] with a false-discovery rate (FDR) of < 10 % [19]. Additionally, we homogenized CEA samples and used the Lipidyzer platform (Sciex, Framingham, MA) [19] and an LTQ Orbitrap XL mass spectrometer (Thermo Fisher Scientific, Bremen, Germany) with an ESI source and MS/MS analysis to further confirm the lipid identification [17].

#### 2.3.2 Data processing

The exported imzML was processed using an in-house developed pipeline [17], executing the following data reduction procedures (1) pre-possessing, smoothing and recalibration of the data using DHB cluster peaks, (2) peak picking using the base peak spectrum, (3) total-ion-current normalization and (4) a fractional mass filter and cross-correlations to remove background *m/z* values and selection of lipid *m/z* values.

### 2.4 Image registration and combined data processing

The tissue section used for μsPA was stained with hematoxylin-eosin and the MALDI-MSI section was stained using Oil Red O. The MALDI-MSI sections were registered to the μsPA image by means of a point-based rigid image registration framework in MeVislab (MeVis Medical Solutions AG, Germany). Per tissue section the Pearson correlation coefficient was calculated between 70 highly abundant *m/z* values and the 1210 nm full slide image taken with μsPA. This was performed on data masked by the plaque area outline, derived from histological segmentation, to ensure the correlation coefficient to be independent from background correlation.

## 3 Results

### 3.1 Micro-spectroscopic PA lipid images compared to MSI

**Fig. 2:**
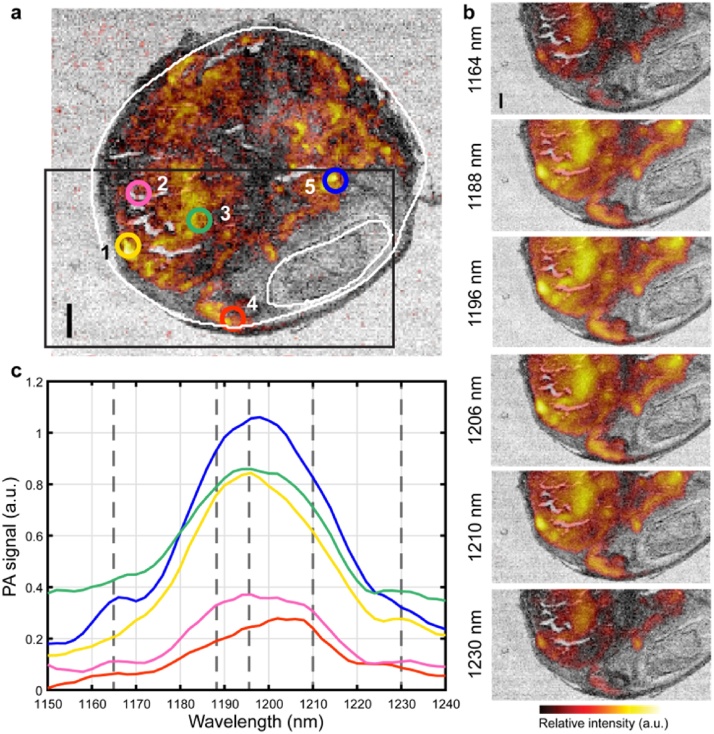
**μsPA measurement** a) μsPA full field of view image at 1210 nm, the black box indicates the ROI, white outlines indicate plaque and lumen b) ROI images showing six wavelengths, 1164, 1188, 1196, 1206, 1210 and 1230 nm, with subtle difference between the images and c) the normalized spectra at five different locations (indicated in figure a) showing differences in the local absorption spectra. Scalebars are 1 mm.

Thirteen tissue sections were imaged using the μsPA system, and an example of such a measurement is shown in Fig. 2. In Fig. 2a, the 1210 nm absorption PA image (in semitransparent red-orange-yellow scale) of the entire section is shown, overlaid on top of the tissue optical transmission image in grayscale. The tissue optical transmission image shows the structure of the tissue section, mapping the optical attenuation of the tissue in the wavelength range under study, whereas the absorption image shows the presence of lipids in the sample. From this image a region of interest was selected, depicted by the black box, and spectral PA imaging was performed within this region, shown in Fig. 2b. To illustrate spectral variations within a sample, spectra from five tissue locations are depicted in Fig. 2c. This shows a variety of spectral shapes present throughout the sample, with local maxima at 1164, 1188, 1196, 1210 and 1230 nm (Fig. 2c, indicated with vertical lines) in locations 5, 2, 1, 4 and 2 (Fig. 2a), respectively.

**Fig. 3:**
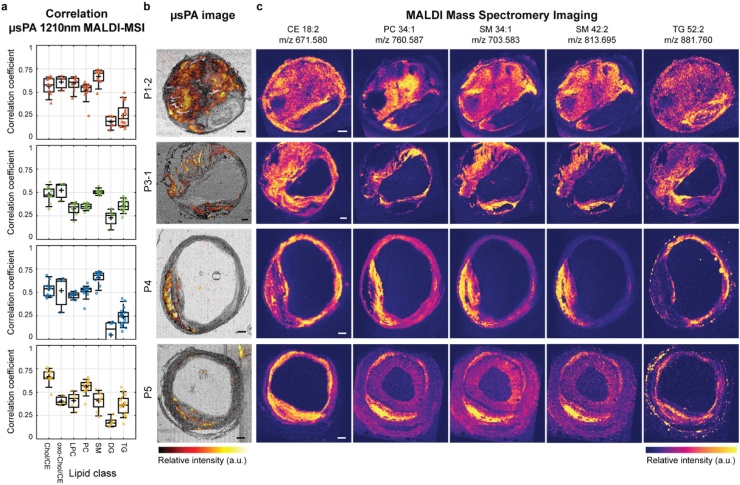
**Correlation of MALDI-MSI to μsPA**. **a**) Pearson correlation of 70 *m/z* MALDI-MSI images to the fullFOV 1210 nm, **b**) Overlay of maximum intensity projection (MIP) of transmission (gray scale) and MIP of absorption (red scale) μsPA image at 1210 nm and **c**) MALDI images showing the spatial distribution of 5 different *m/z* values spanning 4 lipid classes. Two columns, 3 and 4 are both sphingomyelins, showing the same spatial distribution. Scalebars are 1 mm.

The Pearson correlation coefficients between 70 lipids, detected by MALDI-MSI, and the 1210 nm μsPA full slide image was calculated (n = 70), see Fig. 3a. The list of the annotations of all MALDI-MSI detected lipids can be found in Table A1. The correlation with the PA image is generally lowest for diacylglycerol (DG) and triacylglycerol (TG) lipids, and highest for sphingomyelins (SM) and (oxidized) cholesterol and cholesteryl esters (CE).

In Fig. 3b and c, examples of four μsPA images and corresponding MALDI-MSI images are shown. In the analysis of the MALDI-MSI data we see that different lipid classes show the same spatial distribution, *i.e.* SM show the same spatial pattern independent of the tail length [19]. We also found that phosphatidylcholines (PC) are sometimes co-localized with SM, like in P5, see Fig. 3c. However, there are also cases in which there is limited overlap in spatial distribution, as in P3-1 and P4, see Fig. 3c. In cases where co-localization between PC and SM is low, the correlation coefficient of PC between the 1210 nm μsPA image and the MALDI-MSI drops significantly to that of the level of DG and TG, indicating that the signal may not be originating from PC lipids but rather from SM lipids, see Fig. A1 in Supplementary materials.

### 3.2 Analysis of spectral variations

**Fig. 4:**
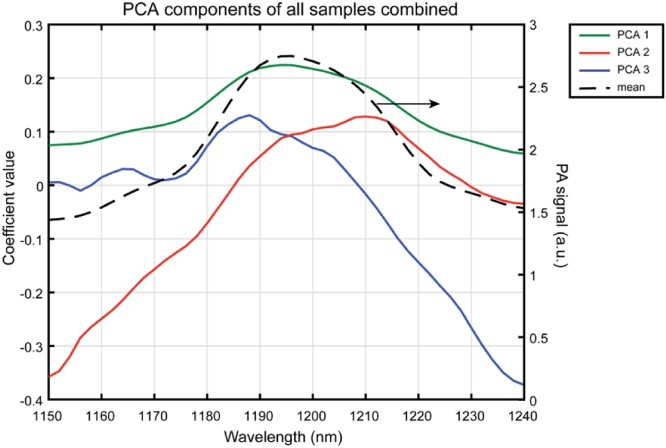
**Spectral information and decomposition of μsPA data**. PCA analysis of all CEA samples simultaneously showing the three spectral components (axis on the left) and the mean PA signal (axis on the right).

Principal component analysis of all samples combined reveals three spectral components to explain the variation in the data, see Fig. 4. The first component has the same shape as the average spectrum, showing a broad peak centered at 1196 nm. The second component shows a peak at 1210 nm and the third component shows two additional peaks at 1164 and 1188 nm.

**Fig. 5:**
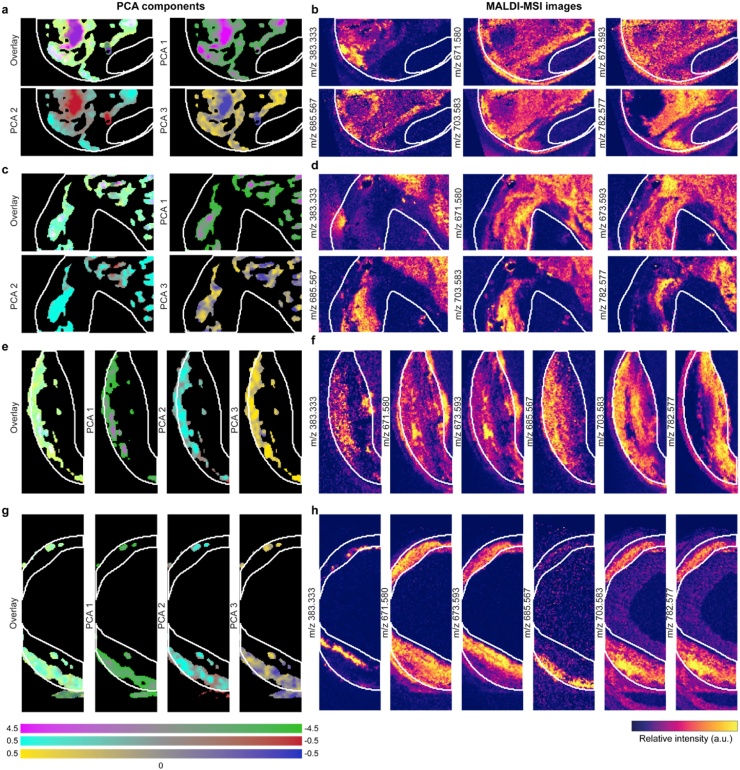
**Spatial distribution of all 3 principal components in and an overlay image, in magenta-green the first principal component, in cyan-red the second principal component and in yellow-blue the third component. Single PCA component images are a maximum intensity projection of the distributions, scaled between -0.5 and 0.5, the overlay images are summed PCA components**, **a-b**) sample P1-2, showing **a**) the spatial distribution of the PCA components and **b)** 6 lipids that imaged by MALDI-MSI, *m/z* 383.333, *m/z* 671.580 (CE 18:2 [M + Na]+), *m/z* 673.593 (CE 18:1 [M + Na]+), *m/z* 685.567 (ox-CE18:2 [M + Na]+), *m/z* 703.583 (SM 34:1 [M+H]+) and *m/z* 782.577 (PC 34:1 [M + Na]+) c-d) sample P3-1, e-f) sample P4 and g-h) sample P5.

These components have different weights per pixel. Fig. 5a, c, e and g show the relative weights of the spectral components in each pixel for selected tissue sections. Magenta, cyan and yellow reflect the positive coefficients per pixel of the PCA components whereas red, green and blue depict the spatial distribution of the negative PCA coefficient value. Spectral variations in PA amplitude reflect the relative abundance of the different lipids throughout the sample (Fig. 5), relative to the mean composition.

MALDI-MSI is a molecular imaging method enabling visualization of the spatial distribution of individual lipid molecules separately. Whereas in μsPA, spectral images correspond to a summation of the absorption of all tissue chromophores at the excitation wavelength, which shall reflect a mixture of the lipids identified by MALDI-MSI present at different concentrations. Therefore, we investigated the spectra of individual lipids to explore possible contributions from different individual lipids towards the complex spectra from the tissue sections. Six lipids from five lipid classes were selected based on highest abundance in the tissue.

**Fig. 6:**
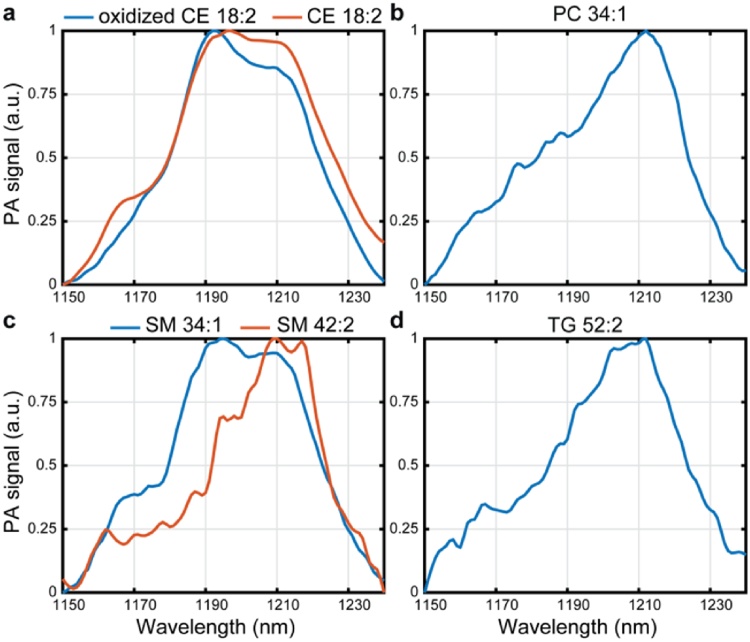
**μsPA recorded spectrum of lipid extracts**. **a**) Cholesteryl linoleate (CE 18:2) in red and oxidized cholersterol linoelate in blue, **b**) Phosphadiatylcholine 16:0/18:1 (PC 34:1), **c**) Sphingomyelin d18:1/16:0 (SM 34:1) in blue and sphingomyelin d18:1/24:1 (SM 42:2) in red and **d**) Triacylglycerol 18:1/18:1/16:0 (TG 52 :2).

Fig. 6 shows the spectra of the pure cholesteryl linoleate (CE 18:2) in normal form and in oxidized form (Fig. 6a), phosphatidylcholine (PC 34:1) (Fig. 6b), sphingomyelins with two different chain lengths (SM 34:1 and SM 42:2) (Fig. 6c) and triacylglycerol (TG 52:2) (Fig. 6d). As expected CE 18:2 and oxidized CE 18:2 have a similar shape, however for the oxidized form the shoulder peak at 1164 nm is missing and the peak at 1212 nm is not as strong. The spectrum of SM 34:1 is similar to that of CE 18:2. Also the spectra of SM 42:2, PC 34:1 and TG 52:2 are comparable in shape, with a main peak at 1210 nm. Interestingly the spectra of both SM lipids differ substantially.

## 4 Discussion

In this study, we explored the imaging lipid vibrations by means of photoacoustic spectroscopy, using a novel PA slide microscope. Carotid endarterectomy cryosections were spectrally imaged using this system revealing vibrational modes generating peaks at or near 1164, 1188, 1196, 1210 and 1230 nm. The μsPA intensity images acquired at 1210 nm showed the strongest correlation with (oxidized) Cholesterol/CEs and SMs in the MALDI-MSI data, lipids that have been found to be abundant in necrotic core plaque. We characterized the variability in the spectral absorption by PCA, which showed three independent components with maxima at 1196 nm (first), 1210 nm (second), and 1164/1188 nm (third component), reflecting the contrast generated by the peak structures in the acquired spectra. As a reference for PA lipid spectra, we imaged pure lipids and found that the relation between molecular structure and the spectral shape of this absorption band is complex. Lipids of composed of differing molecular groups show spectra with high similarity (such as CE 18:2 and SM 34:1) while lipids from the same class, differing only in carbon chain length and saturation may exhibit considerable spectral differences (SM 34:1 and SM 42:2). Since lipid classes tend to spatially cluster in the tissue [19], a direct mapping of atherosclerotic spectral features to molecular composition could not be established.

Based on near-infrared spectroscopic studies, the absorption peaks observed in the spectral range under study mainly result from the second C–H overtone asymmetric stretch (noted as 3${\text{ν}}_{\text{a}}$) molecular vibration [36]; the absorption peaks arising from phosphate and amide groups occur at higher wavelengths [36]. Hydrocarbon chain length and saturation affect the absorption spectra obtained with near-infrared spectroscopy. In particular, the absorption spectra of alkanes, 1-alkenes and aldehydes all exhibit a decrease in the absorption peak ratio of 1190 nm/1210 nm as the hydrocarbon chain length increases; moreover, unsaturated 1-alkenes display a decrease in the absorption peak at 1163 nm in comparison to alkanes [36]. In fact, for longer hydrocarbon chains, the absorption peak at 1210 nm associated with the methylene (CH_2_) group increases and that at 1192 nm, associated with the methyl group (CH_3_), decreases. In addition to the ratio of absorption peaks, slight shifts in the absorption peaks are also observed as the chain length increases. Since lipids that belong to the same lipid class differ only in fatty acid chain length and saturation, we also observed the same spectral shifts phenomena when examining the photoacoustic spectra from the sphingomyelins, as well as those of cholesterol, and cholesteryl esters [29]. Moreover, the observed peaks in the data correspond well with the documented absorption peak ranges in the NIRS studies. The variations associated with the strengths of the methyl, and methylene second overtone absorption peaks are also reflected in the PCA analysis derived from our data set.

Our results may slightly vary under different temperature and pH conditions of the specimens; such changes have been demonstrated to induce shifts ±2 nm in peak position [[36], [37], [38]]. The applied embedding (*vs*. no embedding) and/or fixation (*vs*. no fixation) did not influence the spectral results.

In our previous study using MALDI-MSI only, we discovered that lipids from the same lipid classes show similar spatial distributions [19]. In this study we revealed that, photoacoustically, the spectra of lipids within the same lipid classes, such as SM 34:1 and SM 42:2, are not necessarily the same. Additionally, we found that spectra of different lipid classes, with different spatial distribution within the plaque, can have the same PA spectrum, SM 34:1 and CE 18:2. In fact, the spectra of SM 34:1 and CE 18:2 are so similar that within a tissue sample these would be indistinguishable from each other. Both of these lipids are major contributors to atherosclerosis and have previously been shown to be highly abundant in CEA samples [15,16,19].

Previous publications looking at spectral information to reveal plaque composition mainly focused on the detection of CE 18:1 and CE 18:2 [28,29,39]. The spectra acquired by μsPA are comparable to previously published spectra of cholesteryl esters [29] (Fig. A2 in Supplementary materials). The only difference is in the relative strength of the shoulder peak at 1164 nm.

Considering applicability to *in vivo* imaging, we selected the 1150↙1240 nm second C–H stretch overtone band for the spectroscopic microscopy of atherosclerosis in a tradeoff between optical attenuation and absorption strength. Other absorption bands have also been described and used for PA characterization of plaque [40,41]. The longer-wavelength infrared absorption bands exhibit a larger absorption coefficient and complementary information about molecular structure [36,37,42]. The increased absorption at for instance 1650↙1800 nm may generate a stronger PA signal, but will also limit the penetration depth because of attenuation by other substances such as water. Even at the chosen wavelengths, delivery of sufficient optical power *in vivo* will remain challenging. While a unique mapping between spectral shapes and molecular composition could not be established, the observed variation in spectral absorption is expected to persist in intact specimens and may be related to plaque stage. The relative PA signal strength and peak positions should be investigated in intact specimens and *in vivo* in future studies. Since macroscopic imagers used in those settings have lower spatial resolution, it is possible that the subtle spectral variations we report here may mix and be difficult to discern.

Our setup is, to our best knowledge, the first to allow for spectral imaging of a region of interest, rather than just collecting spectra at pixelwise locations. Indeed, we have shown feasibility of a spectroscopic imaging system allowing simultaneously high spectral and spatial resolution within reasonable scanning times. This system allows for more detailed examination of spectral differences within different regions, and may be applied to other tissue types.

## 5 Conclusions

In conclusion, we have characterized lipid absorption of carotid atherosclerotic plaque at a resolution of 45 μm in the 1150↙1240 nm band. We built a micro-spectroscopic photoacoustic imaging setup with which we spectrally imaged thirteen tissue sections originating from six carotid endarterectomies. Acquired spectra, supplemented by PCA decomposition, revealed four spectral peaks at wavelengths 1164, 1188, 1196, and 1210 nm which reflect most of the spectral variation within and between images. Spectral differences observed were primarily explained by differences in molecular structure such as chain length and number of unsaturated bonds. PA signal intensity was most strongly correlated with sphingomyelin and cholesteryl ester concentration as determined by MALDI-MSI, while diacylglycerols and phosphatidylcholines were associated with lipid-positive but low PA signal areas. The species SM 34:1 and CE 18:2 are abundant in necrotic core and were found to exhibit similar PA spectra, suggesting that this spectral feature can be used for *in vivo* detection of advanced atherosclerotic plaque with features of instability.

## Funding

This work was supported by 10.13039/501100002996Dutch Heart Foundation [project number: NHS2014T096] and 10.13039/501100003246Nederlandse Organisatie voor Wetenschappelijk Onderzoek [project number: 16131].

## Declaration of Competing Interest

The authors declare that there are no conflicts of interest.
