# Supplementary material for: Micro Spectroscopic Photoacoustic (μsPA) imaging of advanced carotid atherosclerosis
Source: Photoacoustics. 2021 Mar 18;22:100261. doi: 10.1016/j.pacs.2021.100261 (PMC8027769; doi:10.1016/j.pacs.2021.100261)
Supplement: Supplementary file 1 [file mmc1.docx]

**Appendices**

**Micro Spectroscopic Photoacoustic (µsPA) imaging of advanced carotid atherosclerosis**

Sophinese Iskander-Rizk^1*^, Mirjam Visscher^1*^, Astrid Moerman^1^, Suze-Anne Korteland^1^, Kim van der Heiden^1^, Antonius F.W. van der Steen^1^ and Gijs van Soest^1^

^1^ Department of Cardiology, Erasmus MC University Medical Center Rotterdam, Rotterdam, The Netherlands

^*^*These authors equally contributed to this work*

**Table A1:** List of 70 lipids identified/assigned in human carotid atherosclerotic plaques

**Figure A1:** Example of sample where PC and SM are mutually exclusive in terms of spatial distribution

**Figure A2:** Comparison of CE18:2 PA signal

**Table A2:** P-values correlation coefficient µsPA compared to MALDI-MSI

**Table A1.** List of 70 lipids identified/assigned in human carotid atherosclerotic plaques

| m/z ± 0.02 | lipid class | ID | adduct | m/z ± 0.02 | lipid class | ID | adduct |
| --- | --- | --- | --- | --- | --- | --- | --- |
| 369.350* | Chol | Cholesterol^c^ | [M-H2O+H]+ | 756.558* | PC | PC(32:0)^a,b^ | [M+Na]+ |
| 401.343* | oxChol | 7-ketocholestol^d^ | [M+H]+ | 758.574* | PC | PC(34:2)^a,c^ | [M+H]+ |
| 496.346* | LPC | LPC(16:0)^d^ | [M+H]+ | 760.587* | PC | PC(34:1)^a,b,c^ | [M+H]+ |
| 518.335* | LPC | LPC(18:3)^d^ | [M+H]+ | 766.578* | PC-O | PC(O-36:5) ^LM,HMDB,b^ | [M+H]+ |
| 520.351* | LPC | LPC(18:2)^d^ | [M+H]+ | 768.581* | PC-O | PC(O-36:4) ^LM,HMDB,b^ | [M+H]+ |
| 522.371* | LPC | LPC(18:1)^d^ | [M+H]+ | 780.564* | PC | PC(34:2)^a,b,c^ | [M+Na]+ |
| 524.374* | LPC | LPC(18:0)^d^ | [M+H]+ | 782.577* | PC | PC(36:4)^a,c^  PC(34:1)^a,b^ | [M+H]+  [M+Na]+ |
| 542.322* | LPC | LPC(20:5)^d^ | [M+H]+ | 784.591* | PC | PC(36:3)^a,b,c^ | [M+H]+ |
| 544.340* | LPC | LPC(20:4)^d^ | [M+H]+ | 786.610* | PC | PC(36:2)^a,b,c^ | [M+H]+ |
| 546.351* | LPC | LPC(20:3)^d^  LPC(18:0)^b^ | [M+H]+  [M+Na]+ | 788.620* | PC | PC(36:1)^c^ | [M+H]+ |
| 549.488* | DG | DG(32:1)^a^ | [M-H2O+H]+ | 807.641* | SM | SM(40:2)^a,b^ | [M+Na]+ |
| 575.510* | DG | DG(34:2)^a^  DG(O-32:1)^b^ | [M-H2O+H]+  [M+Na]+ | 810.610* | PC | PC(38:4)^b,c^ | [M+H]+ |
| 577.520* | DG | DG(34:1)^a^  DG(O-32:0)^b^ | [M-H2O+H]+  [M+Na]+ | 811.676* | SM | SM(40:0)^b^ | [M+Na]+ |
| 599.497* | DG | DG(36:4)^a^ | [M-H2O+H]+ | 813.695* | SM | SM(42:2)^a,b,c^ | [M+H]+ |
| 601.512* | DG | DG(36:3)^a^  DG(O-34:2)^b^ | [M-H2O+H]+  [M+Na]+ | 820.539* | PC | PC(36:4)^a^ | [M+K]+ |
| 603.530* | DG | DG(36:2)^a^  DG(O-34:1)^b^ | [M-H2O+H]+  [M+Na]+ | 827.709* | TG | TG(48:1)^a^ | [M+Na]+ |
| 645.561* | CE | CE(16:1)^a,b,c^ | [M+Na]+ | 829.717* | TG | TG(48:0)^a^ | [M+Na]+ |
| 647.577* | CE | CE(16:0)^a,b,c^ | [M+Na]+ | 835.673* | SM | SM(44:5)^b^  SM(42:2) ^a,b^ | [M+H]+ [M+Na]+ |
| 671.580* | CE | CE(18:2)^a,b,c^ | [M+Na]+ | 853.721* | TG | TG(50:2)^a,b^ | [M+Na]+ |
| 673.593* | CE | CE(18:1)^a,b,c^ | [M+Na]+ | 857.750* | TG | TG(50:0)^a^ | [M+Na]+ |
| 685.567* | oxCE | Oxo-ODE-CE^d^ | [M+Na]+ | 877.738* | TG | TG(52:4)^a,b^ | [M+Na]+ |
| 687.566* | oxCE*  CE | HODE-CE^b^  CE(18:2)^a^ | [M+Na]+  [M+K]+ | 879.738* | TG | TG(52:3)^a,b^ | [M+Na]+ |
| 693.567* | CE | CE(20:5)^a,b^ | [M+Na]+ | 881.760* | TG | TG(52:2)^a,b^ | [M+Na]+ |
| 695.581* | CE | CE(20:4)^a,b^ | [M+Na]+ | 883.768* | TG | TG(52:1)^a,b^ | [M+Na]+ |
| 697.535* | SM | SM(32:1)^a,b^ | [M+Na]+ | 885.781* | TG | TG(52:0)^a^ | [M+Na]+ |
| 697.591* | CE | CE(20:3)^a,b^ | [M+Na]+ | 901.732* | TG | TG(54:6)^a,b^ | [M+Na]+ |
| 703.583* | SM | SM(34:1)^a,b,c^ | [M+H]+ | 903.751* | TG | TG(54:5)^a,b^ | [M+Na]+ |
| 711.550* | oxCE*  CE | CE(11:1D3)^HMDB^  CE(20:4)^a^ | [M+Na]+  [M+K]+ | 905.751* | TG | TG(54:4)^a,b^ | [M+Na]+ |
| 719.571* | CE | CE(22:6)^a,b^ | [M+Na]+ | 907.773* | TG | TG(54:3)^a,b^ | [M+Na]+ |
| 721.586* | CE | CE(22:5)^a,b,c^ | [M+Na]+ | 909.794* | TG | TG(54:2)^a,b^ | [M+Na]+ |
| 725.565* | SM | SM(34:1)^a,b,c^ | [M+Na]+ | 927.744* | TG | TG(56:7)^a^ | [M+Na]+ |
| 729.590* | SM | SM(36:2)^a,b^ | [M+H]+ | 929.768* | TG | TG(56:6)^a,b^ | [M+Na]+ |
| 731.614* | SM | SM(36:1)^a,b,c^ | [M+H]+ | 931.778* | TG | TG(56:5)^a,b^ | [M+Na]+ |
| 734.580* | PC | PC(32:0)^a,b,c^ | [M+H]+ | 933.790* | TG | TG(56:4)^a^ | [M+Na]+ |
| 741.545* | SM | SM(34:1)^a,b^ | [M+K]+ | 955.774* | TG | TG(58:7)^a^ | [M+Na]+ |

*m/z = mass measured in MALDI-MSI experiment using Synapt G2Si TOF system. For exact mass measured with FTICR and ppm values, we refer to the online available METASPACE at https://metaspace2020.eu/ data named Human CEA Patient H - section 4 and Human CEA Patient I - section 4*

*lipid group = assigned lipid group based on database search, i.e. LipidMaps, HMDB.*

*ID = proven lipid identity, superscript denotes identification method: a. Lipidyzer MRM analysis, b. FTICR measurement combined with METASPACE database (FDR of maximum 10%), c. Identified in previous study(19), d. Identified from literature, LM: assigned from search in LipidMaps, HMDB: assigned from search in HMDB*

*adduct = positive ion adduct in identification experiment.*

** Asterisks denote the lipids that were included in the cross-correlation analysis*

^d^ *Lipid identity not confirmed in identification experiments due to methodological limitations. However m/z values have been identified as 7-ketocholesterol(14,61), other cholesterol derivatives(61), LPCs(62) and oxo-ODE-CE(12) in literature and have been included as such in our cross-correlation analysis.*


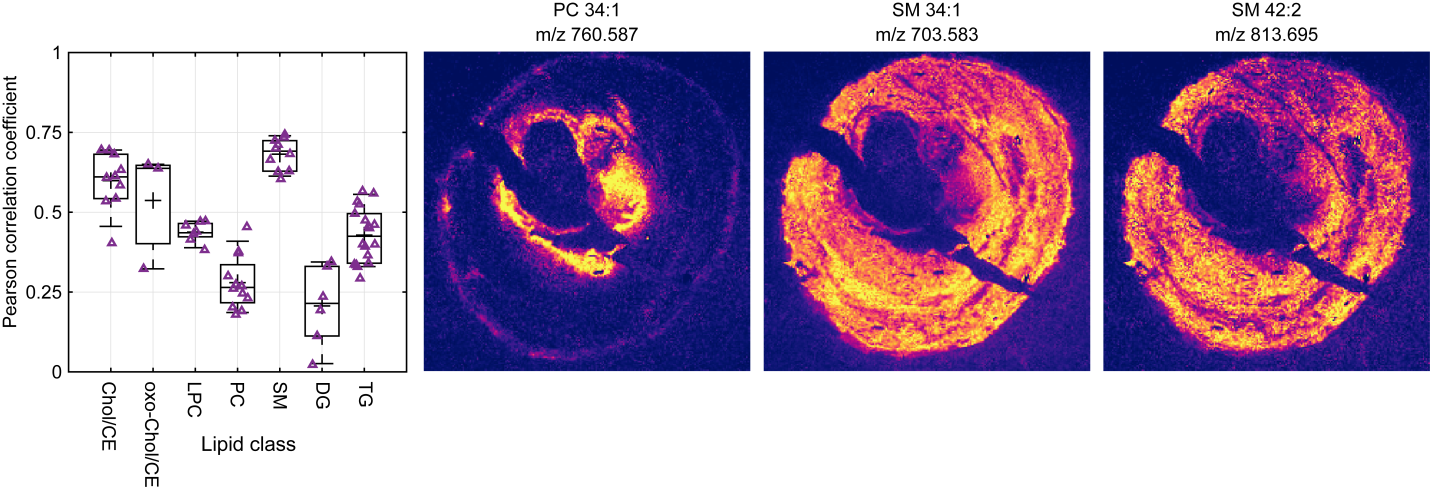


**Figure A1:** Example of sample where PC and SM are mutually exclusive in terms of spatial distribution P2-2

**
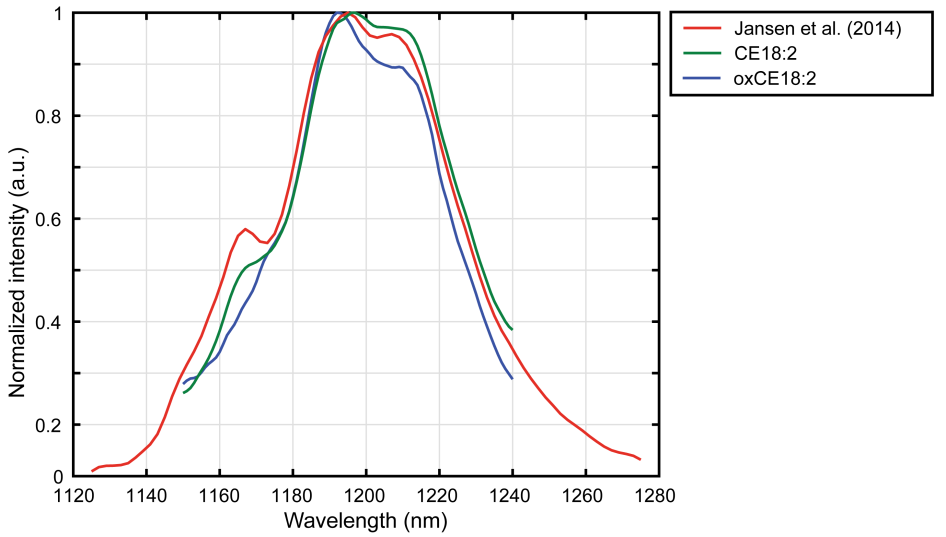
**

**Figure A2: Comparison of CE18:2 PA signal** measured by K. Jansen et.al (2014) to the measurement of CE 18:2 and oxCE18:2 by µsPA.

**Table A2:** P-values correlation coefficient µsPA compared to MALDI-MSI, significant p-value* < 0.05

**Sample P1-1**

|  | Chol/CE | oxo-Chol/CE | LPC | PC | SM | DG | TG |
| --- | --- | --- | --- | --- | --- | --- | --- |
| Chol/CE |  | 0.9371 | 0.2295 | 0.1003 | *0.0036 | *0.0002 | *0.0001 |
| Oxo-Chol/CE |  |  | 0.6667 | 0.4393 | 0.0769 | 0.0952 | 0.2926 |
| LPC |  |  |  | *0.0020 | *0.0004 | *0.0012 | *0.0026 |
| PC |  |  |  |  | *0.0120 | *0.0001 | *<0.0001 |
| SM |  |  |  |  |  | *0.0002 | *<0.0001 |
| DG |  |  |  |  |  |  | *0.0046 |
| TG |  |  |  |  |  |  |  |

**Sample P1-2**

|  | Chol/CE | oxo-Chol/CE | LPC | PC | SM | DG | TG |
| --- | --- | --- | --- | --- | --- | --- | --- |
| Chol/CE |  | 0.4685 | 0.5726 | 0.2265 | *0.0140 | *0.0002 | *0.0001 |
| Oxo-Chol/CE |  |  | 0.6303 | 0.1893 | 0.2867 | *0.0238 | *0.0081 |
| LPC |  |  |  | *0.0390 | *0.0434 | *0.0006 | *0.0001 |
| PC |  |  |  |  | *0.0009 | *0.0001 | *<0.0001 |
| SM |  |  |  |  |  | *0.0002 | *<0.0001 |
| DG |  |  |  |  |  |  | 0.3446 |
| TG |  |  |  |  |  |  |  |

**Sample P1-3**

|  | Chol/CE | oxo-Chol/CE | LPC | PC | SM | DG | TG |
| --- | --- | --- | --- | --- | --- | --- | --- |
| Chol/CE |  | 0.6593 | *0.0032 | *0.0001 | *0.0102 | *0.0002 | *0.0009 |
| Oxo-Chol/CE |  |  | 0.0714 | *0.0071 | 0.1608 | *0.0238 | 0.0850 |
| LPC |  |  |  | 0.9241 | 0.1292 | 1 | 0.8311 |
| PC |  |  |  |  | *0.0101 | 0.7012 | 0.7298 |
| SM |  |  |  |  |  | *0.0160 | 0.1617 |
| DG |  |  |  |  |  |  | 0.5455 |
| TG |  |  |  |  |  |  |  |

**Sample P2-1**

|  | Chol/CE | oxo-Chol/CE | LPC | PC | SM | DG | TG |
| --- | --- | --- | --- | --- | --- | --- | --- |
| Chol/CE |  | 0.9371 | *0.0117 | *0.0002 | *<0.0001 | 0.9578 | 0.3709 |
| Oxo-Chol/CE |  |  | 0.2788 | *0.0143 | *0.0091 | 0.3810 | 0.5030 |
| LPC |  |  |  | 0.0648 | *0.0111 | *0.0013 | *0.0045 |
| PC |  |  |  |  | 0.0615 | *0.0001 | *<0.0001 |
| SM |  |  |  |  |  | *0.0004 | *<0.0001 |
| DG |  |  |  |  |  |  | *0.0330 |
| TG |  |  |  |  |  |  |  |

**Sample P2-2**

|  | Chol/CE | oxo-Chol/CE | LPC | PC | SM | DG | TG |
| --- | --- | --- | --- | --- | --- | --- | --- |
| Chol/CE |  | 0.9371 | *0.0021 | *0.0001 | *0.0211 | *0.0002 | *0.0002 |
| Oxo-Chol/CE |  |  | 0.4970 | *0.0308 | 0.1608 | 0.0952 | 0.3397 |
| LPC |  |  |  | *0.0010 | *<0.0001 | *0.0007 | 0.8026 |
| PC |  |  |  |  | *0.0001 | 0.2908 | *0.0003 |
| SM |  |  |  |  |  | *0.0002 | *<0.0001 |
| DG |  |  |  |  |  |  | *0.0019 |
| TG |  |  |  |  |  |  |  |

**Sample P2-3**

|  | Chol/CE | oxo-Chol/CE | LPC | PC | SM | DG | TG |
| --- | --- | --- | --- | --- | --- | --- | --- |
| Chol/CE |  | 0.2867 | *0.0031 | 0.3068 | *0.0008 | *0.0047 | *0.0747 |
| Oxo-Chol/CE |  |  | 0.0848 | 0.4484 | *0.0280 | *0.0357 | *0.0443 |
| LPC |  |  |  | *0.0008 | 0.1457 | *0.0016 | *0.0001 |
| PC |  |  |  |  | *0.0002 | *0.0039 | *0.0037 |
| SM |  |  |  |  |  | *0.0006 | *<0.0001 |
| DG |  |  |  |  |  |  | 0.0848 |
| TG |  |  |  |  |  |  |  |

**Sample P3-1**

|  | Chol/CE | oxo-Chol/CE | LPC | PC | SM | DG | TG |
| --- | --- | --- | --- | --- | --- | --- | --- |
| Chol/CE |  | 0.4560 | *0.0012 | *0.0015 | 0.5035 | *0.0003 | *0.0009 |
| Oxo-Chol/CE |  |  | *0.0121 | *0.0044 | 0.4685 | *0.0238 | *0.0172 |
| LPC |  |  |  | 0.7285 | *<0.0001 | *0.0293 | 0.3171 |
| PC |  |  |  |  | *0.0001 | *0.0004 | 0.5747 |
| SM |  |  |  |  |  | *0.0002 | *<0.0001 |
| DG |  |  |  |  |  |  | *0.0035 |
| TG |  |  |  |  |  |  |  |

**Sample P3-2**

|  | Chol/CE | oxo-Chol/CE | LPC | PC | SM | DG | TG |
| --- | --- | --- | --- | --- | --- | --- | --- |
| Chol/CE |  | 0.7692 | *<0.0001 | *<0.0001 | 0.8053 | *0.0001 | *<0.0001 |
| Oxo-Chol/CE |  |  | *0.0121 | *0.0036 | 0.9371 | *0.0238 | *0.0077 |
| LPC |  |  |  | *0.0013 | *<0.0001 | *0.0007 | *0.0020 |
| PC |  |  |  |  | *0.0001 | *0.0009 | 0.1333 |
| SM |  |  |  |  |  | *0.0002 | *<0.0001 |
| DG |  |  |  |  |  |  | *0.0005 |
| TG |  |  |  |  |  |  |  |

**Sample P3-3**

|  | Chol/CE | oxo-Chol/CE | LPC | PC | SM | DG | TG |
| --- | --- | --- | --- | --- | --- | --- | --- |
| Chol/CE |  | *0.0385 | *0.0025 | *<0.0001 | 0.8053 | *0.0003 | *0.0002 |
| Oxo-Chol/CE |  |  | *0.0121 | *0.0036 | *0.0490 | *0.0238 | *0.0074 |
| LPC |  |  |  | *0.0002 | *<0.0001 | *0.0047 | 0.2764 |
| PC |  |  |  |  | *0.0001 | *0.0220 | *<0.0001 |
| SM |  |  |  |  |  | *0.0002 | *<0.0001 |
| DG |  |  |  |  |  |  | 0.1522 |
| TG |  |  |  |  |  |  |  |

**Sample P4**

|  | Chol/CE | oxo-Chol/CE | LPC | PC | SM | DG | TG |
| --- | --- | --- | --- | --- | --- | --- | --- |
| Chol/CE |  | 0.8112 | 0.0831 | 0.7802 | *0.0073 | *0.0002 | *<0.0001 |
| Oxo-Chol/CE |  |  | 0.4970 | 0.4393 | 0.1119 | *0.0238 | *0.0237 |
| LPC |  |  |  | *0.0101 | *0.0003 | *0.0007 | *0.0001 |
| PC |  |  |  |  | *0.0011 | *0.0001 | *<0.0001 |
| SM |  |  |  |  |  | *0.0002 | *<0.0001 |
| DG |  |  |  |  |  |  | *0.0085 |
| TG |  |  |  |  |  |  |  |

**Sample P5**

|  | Chol/CE | oxo-Chol/CE | LPC | PC | SM | DG | TG |
| --- | --- | --- | --- | --- | --- | --- | --- |
| Chol/CE |  | *0.0055 | *0.0009 | *0.0018 | *0.0002 | *0.0002 | *<0.0001 |
| Oxo-Chol/CE |  |  | 1 | *0.014 3 | 0.6303 | *0.0238 | 0.4510 |
| LPC |  |  |  | *0.0044 | 0.9433 | *0.0043 | 0.3515 |
| PC |  |  |  |  | *0.0021 | *0.0001 | *<0.0001 |
| SM |  |  |  |  |  | *0.0013 | 0.2113 |
| DG |  |  |  |  |  |  | *0.0019 |
| TG |  |  |  |  |  |  |  |

**Sample P6**

|  | Chol/CE | oxo-Chol/CE | LPC | PC | SM | DG | TG |
| --- | --- | --- | --- | --- | --- | --- | --- |
| Chol/CE |  | *0.0055 | *0.0068 | *<0.0001 | 0.1774 | 0.3011 | 0.9657 |
| Oxo-Chol/CE |  |  | 0.0848 | *0.0036 | *0.0121 | *0.0952 | *0.0098 |
| LPC |  |  |  | *0.0002 | *0.0019 | 0.2824 | *0.0409 |
| PC |  |  |  |  | *0.0016 | *0.0001 | *0.0007 |
| SM |  |  |  |  |  | *0.0426 | 0.3810 |
| DG |  |  |  |  |  |  | 0.4643 |
| TG |  |  |  |  |  |  |  |
